# Supplementary material for: Outcomes of Patients with Metastatic Castration-Resistant Prostate Cancer According to Somatic Damage DNA Repair Gene Alterations
Source: Curr Oncol. 2022 Apr 15;29(4):2776–91. doi: 10.3390/curroncol29040226 (PMC9030073; doi:10.3390/curroncol29040226)
Supplement: Supplementary file 1 [file curroncol-29-00226-s001.zip › curroncol-1654807-supplementary-xml.pdf]

SUPPLEMENTARY DATA

Gènes
ARID1A, ARID1B, ATM, ATR, ATRX, AURKA, BAP1, BARD1, BLM, BRCA1, BRCA2, BRCC3, BRIP1, CDK12, CHEK1, CHEK2, EMSY, ERCC1, FAM175A, FANCA, FANCB, FANCC, FANCD2, FANC, FANCF, FANCG, FANCL, FANCI, H2AFX, HDAC1, HDAC2, HDAC, MAD2L2, MDC1, MERIT40, MRE11A, NBN, PALB2, PARP1, RAD17, RAD50, RAD51B, RAD51B, RAD51, RPA1, SHFM1, SLX4, SMARCA2, SMARCA4, TDG, TOPBP1, TP53BP1, XPA, XRCC2, XRCC3 et XRCC4

Table S1 - Lists of genes in the panel and methology

The somatic analyzes were performed using DNA extracted from the initial biopsy or surgical excision, in the Laboratory of Biology and Genetics of the François Baclesse Center (U1245). The DNA used to prepare the NGS libraries was extracted from formaldehyde-embedded fixed tissue samples (FFPE) (QIAgen Qiamp DNA FFPE Tissue Kit). A capture-based enrichment process (SureSelect XT Custom Design, Agilent) was performed by targeting coding sequences (+/- 25 bp) of a custom panel of 65 genes involved in the DNA repair pathway (***Table S3***). The libraries were sequenced on the Illumina NextSeq sequencer and the NGS data were analyzed using a custom bioinformatics analysis detecting the nucleic bases. The quality criteria required for the analysis were defined as follows: (i) a minimum coverage at each base of 200X for more than 95% of the region of interest; (ii) a minimal mutated allele fraction of 5%. Only pathogenic or possibly pathogenic variants resulting in truncating protein truncation alterations (nonsense, frame shift insertions and deletions, and splice site mutations) or missense variants compatible with pathogenicity have been reported, while variants of unknown significance were excluded. The term somatic alteration concerns in this study the pathogenic variations identified from somatic analyzes. It does not determine in any case the origin of the alteration, that is to say, germline or pure somatic.

		mCRPC included		n	mCRPC excluded		n	p
**INITIAL PSA**		28,8	[1.3-5500]	83	24,5	[1.32-5000]	136	0.71
**GLEASON SCORE**				82			136	0.01
4 à 7		35	(43%)		83	(61%)		
8 à 10		47	(57%)		53	(39%)		
**TNM**				83			149	0.46
T1/2		21	(25%)		48	(32%)		
T3/4		52	(63%)		81	(54%)		
TX		10	(12%)		20	(13%)		
N0		16	(19%)		62	(42%)		0.001
N1+		25	(30%)		25	(17%)		
Nx		42	(51%)		62	(42%)		
M0/MX		40	(48%)		87	(58%)		0.17
M1		43	(52%)		62	(42%)		
**ECOG**				78			120	0.12
	0	34	(44%)		47	(39%)		
	1	38	(49%)		51	(43%)		
	2	6	(8%)		16	(13%)		
	3	0	(0%)		6	(5%)		
**PREVIOUS TREATMENTS**								
Surgery		22	(27%)	82	37	(25%)	148	0.88
Chemotherapy		14	(17%)	83	10	(7%)	148	0.028
Hormonotherapy		82	(100%)	82	148	(99%)	150	0.54
Prostate radiotherapy		33	(73%)	45	70	(47%)	148	0.004
Pelvis radiotherapy		28	(62%)	45	48	(32%)	148	0.001
**FIRST-LINE TREATMENTS**				83			138	0.95
Abiraterone		39	(47%)		67	(49%)		
Taxanes		19	(23%)		29	(21%)		
Enzalutamide		25	(30%)		42	(30%)		
**TREATMENT LINES AFTER CASTRATION RESISTANCE**		2	[1-5]		2	[1-5]		0.54
**DURATION OF HORMONOSENSITIVITY**		2,1	[0.4-18.1]		5,77	[0-81.8]		0.001
**TIME BEFORE METASTASIS**		0,04	[0-13.8]		2,1	[0-81.8]		0.05

Table S2– Outcomes of analyzed *versus* excluded patients.
BMI: body mass index; DDR+: mutated patients, DDR- non-mutated patients; n: number of patients; PSA: prostate specific antigen; NHT: new generation hormonotherapy; PSA: prostate specific antigen.

	Total		NGHT		TAXANES		p
**AGE**	69.5 [23-82]	83	69.6 69.21	70	69 '(5.61)	64	0.77
**ECOG**		78		59		19	0.78
0	34 (44%)		27 (46%)		7 (37%)		
1	38 (49%)		27 (46%)		11 (58%)		NA
2	6 (8%)		5 (8%)		1 (5%)		NA
**BMI**	27.5 [23-38]	74	28 [23-38.3]	56	27 [25-29.3]	18	0.35
**PREVIOUS TREATMENTS**				34		13	
Surgery	22 (27%)	82	17 (27%)		5 (28%)		1
Chemotherapy	14 (17%)	83	13 (20%)		1 (5%)		0.17
Radiotherapy	45 (54%)	83	33 (52%)		12 (63%)		0.53
**DDR ALTERATIONS**		83		64		19	0.7
DDR+	33 '(47%)		24 (33%)		9 (47%)		
DDR-	50 '(30.1%)		40 (67%)		10 (53%)		NA
**GLEASON SCORE**		82		63		18	0.21
5 to 7	35 '(42.7%)		24 (38%)		11 (58%)		
8 to 10	47 '(57.3%)		39 (62%)		8 (42%)		NA
**TNM**		83		31		18	0.93
T1/2	42 (51%)		17 (67%)		4 (21%)		
T3/4	41 (49%)		39 (61%)		13 (68%)		NA
Tx	10 (12%)		8 (12%)		2 (11%)		NA
N1+	25 (30%)		20 (31%)		5 (27%)		0.67
N0	16 (19%)		11 (17%)		5 (27%)		NA
Nx	42 (51%)		33 (52%)		9 (47%)		NA
M1	43 (52%)		34 (53%)		9 (47%)		0.86
M0/Mx	40 (48%)		30 (47%)		10 (53%)		NA
**INITIAL PSA**	28.8 [1.3-5500]	83	32.5 [1.27-5000]	31	20.5 [2.2-459]	18	0.58
**DIAGNOSTIC MODES**		79		28		17	1
Symptoms	49 (62)%		15 '(53.6%)		10 '(58.8%)		
Individual screening	30 (38%)		13 '(46.4%)		7 '(41.2%)		NA
**LINES OF TREATMENTS**		33		10		10	0.89
1	8 (24.2%)		2 (22%)		3 (30%)		
2	14 (42.4%)		3 (39%)		5 (50%)		NA
3	7 (21.2%)		3 (26%)		1 (10%)		NA
4	3 (9.1%)		1 (9%)		1 (10%)		NA
5	1 (3%)		1 (4%)		0 (0%)		NA

Table S3– Clinical outcomes of analyzed patients according to treatments (NHT *versus* Taxanes)

BMI: body mass index; DDR+: mutated patients, DDR- non-mutated patients; n: number of patients; PSA: prostate specific antigen; NHT: new generation hormonotherapy; PSA: prostate specific antigen.

File S1 - Annex of molecular results

Seventy-nine tumor analyzes were performed with a panel of 69 DDR genes. Two other analyzes were performed with smaller panels for 11 or 15 DDR genes: no somatic mutation was identified in these two samples. One patient had a germline BRCA2 mutation: somatic analysis was not performed. A last patient had benefited from a somatic analysis as part of a therapeutic trial, identifying a *CHEK2* mutation that is also present on the 69 gene panel. Somatic analysis on 69-gene panel was not repeated.

In terms of quality criterion, 3 analyzes did not reach a maximum coverage of 100% (95%, 90% and 73%): these analyzes did not identify mutations. Five tumor samples had less than 30% of tumor cells and four of them did not have DDR mutations.

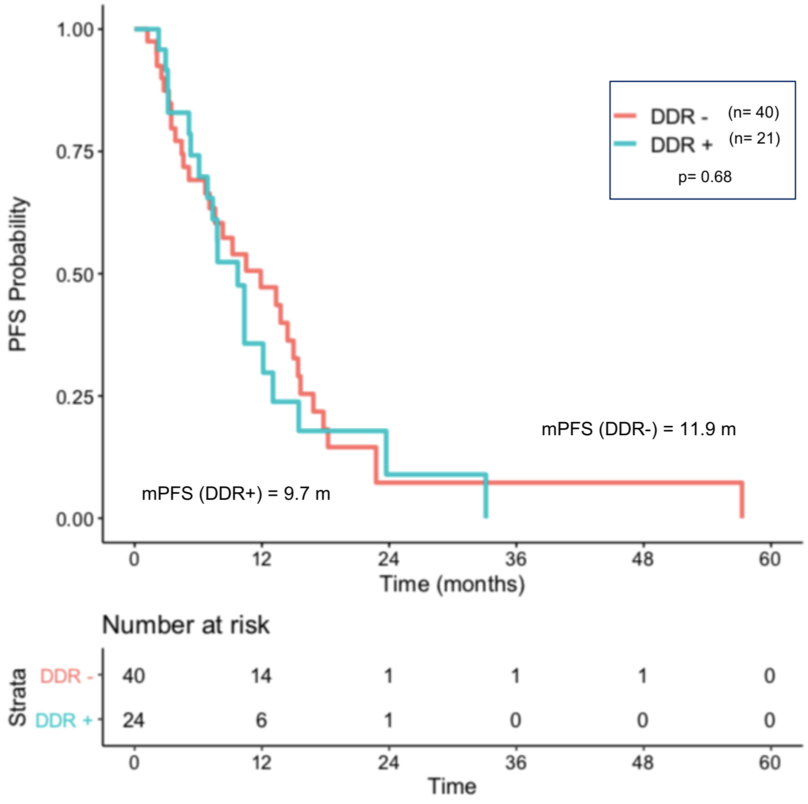

Figure S1 – First-line treatment mCRPC PFS according to somatic DDR+ *versus* DDR- alterations among patients who received NHT .

DDR+: mutated patients, DDR- non-mutated patients; m: months; mCRPC: metastatic castration-resistant prostate cancer; mPFS: median progression-free survival; n: number of patients

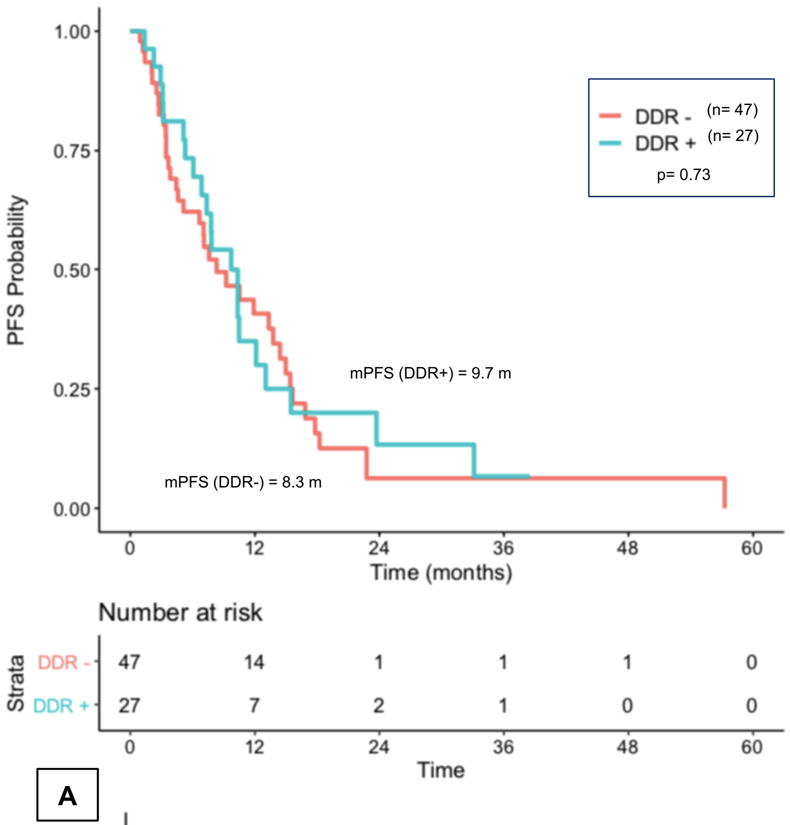

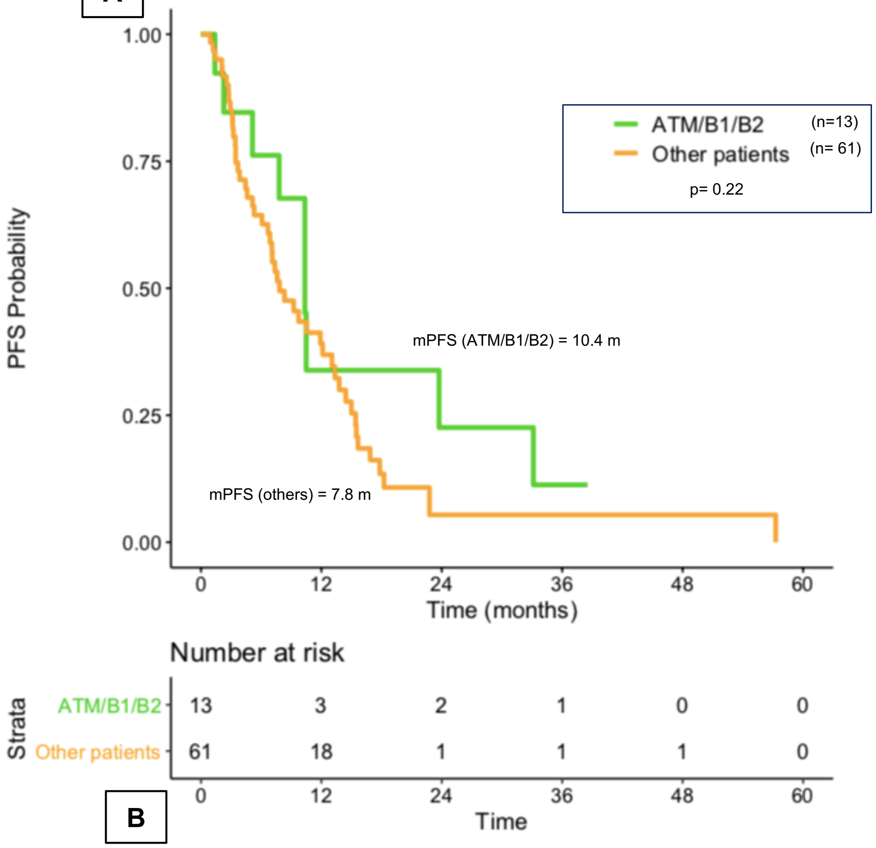

Figure S2– First exposure to NHT among mCRPC patients in first two lines according to somatic DDR+ versus DDR- alterations and (A) according to ATM/B1/B2 mutated patients versus other patients (B).
DDR+: mutated patients, DDR- non mutated patients; m: months; mCRPC: metastatic castration-resistant prostate cancer; median PFS: median progression-free survival; n: number of patients

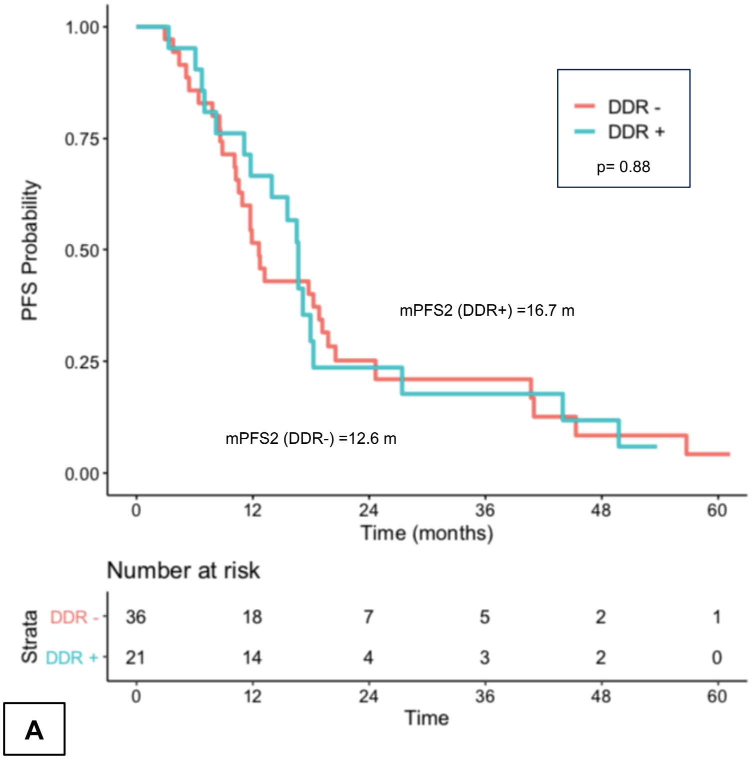

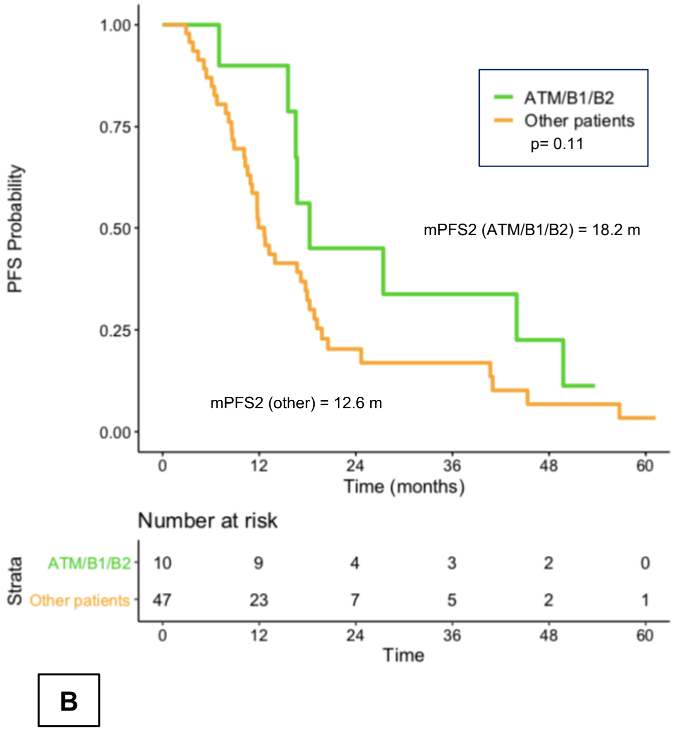

Figure S3 – PFS2 according to somatic DDR+ *versus* DDR- alterations (A) according to ATM/B1/B2-mutated patients *versus* other patients (B), according to somatic DDR+ *versus* DDR- .

CHS: chemptherapy followed by NHGT sequence; DDR+: mutated patients, DDR- non-mutated patients; HCS: NHT followed by chemotherapy sequence m: months; mCRPC: metastatic castration-resistant prostate cancer; median PFS: median progression-free survival; n: number of patients

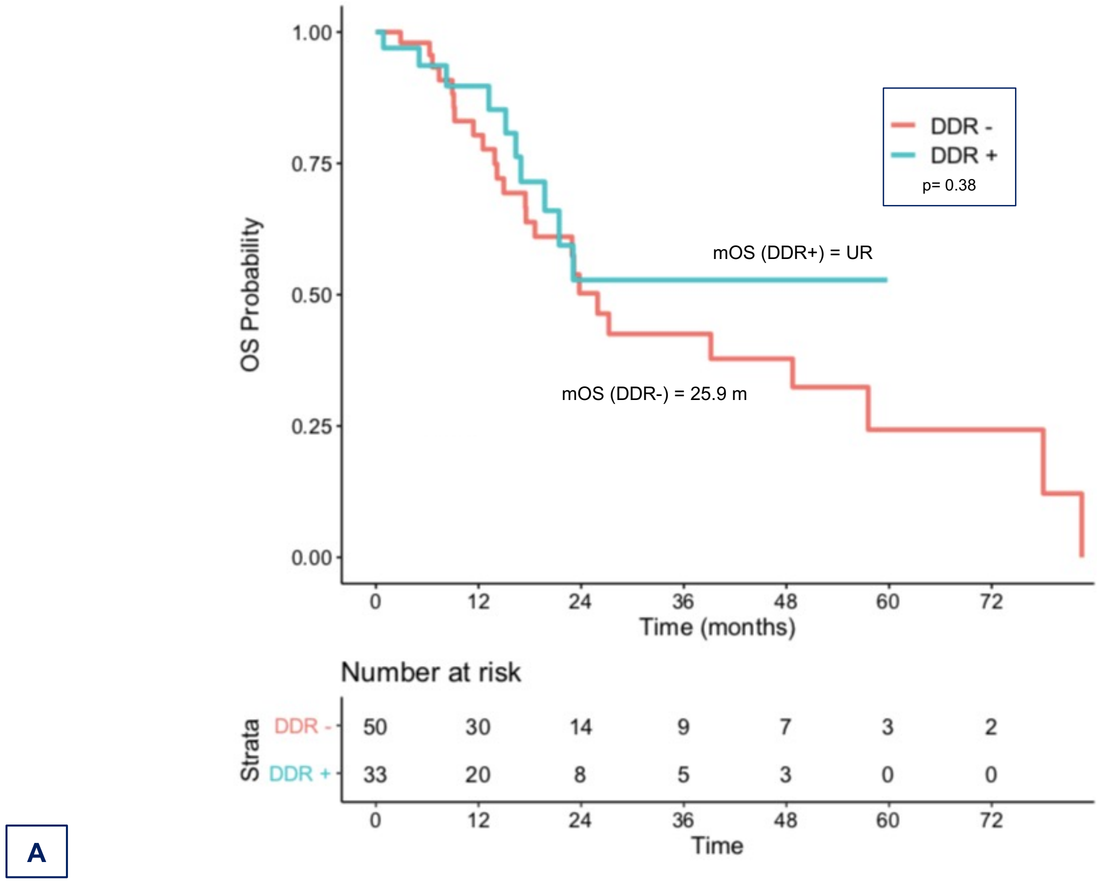

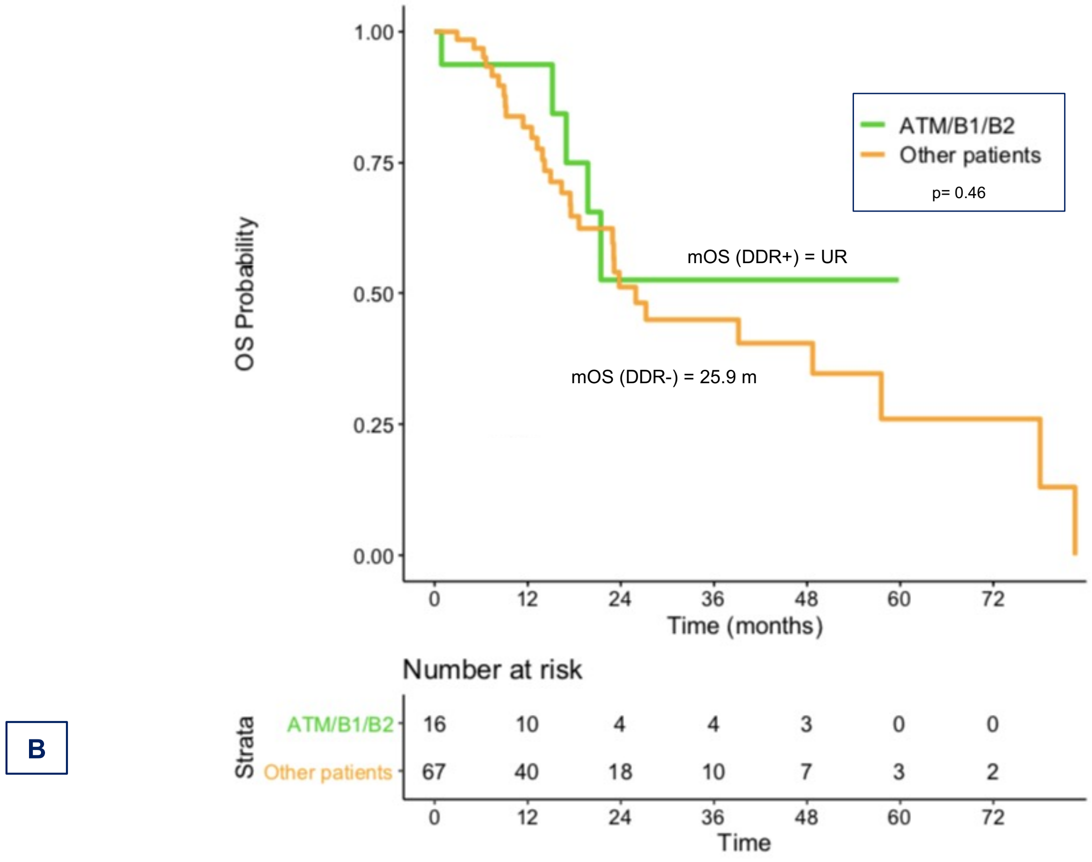

Figure S4– OS according to somatic DDR+ *versus* DDR- alterations (A) and according to ATM/B1/B2-mutated patient *versus* the other patients (B).
DDR+: mutated patients, DDR- non mutated patients; m: months; mCRPC: metastatic castration (resistant prostate cancer; mOS: median overall survival
